# Study invitations with envelopes made from recycled paper do not increase likelihood of active responses or study participation in the German National Cohort

**DOI:** 10.1186/s13104-019-4510-y

**Published:** 2019-07-31

**Authors:** Malte Langeheine, Hermann Pohlabeln, Wolfgang Ahrens, Kathrin Günther, Stefan Rach

**Affiliations:** 1 Leibniz Institute for Prevention Research and Epidemiology-BIPS, Achterstrasse 30, 28359 Bremen, Germany; 2 Institute of Statistics, Faculty of Mathematics and Computer Science, University Bremen, P.O. Box 330 440, 28334 Bremen, Germany

**Keywords:** Study participation, Trial, Recycled envelopes, German National Cohort, Paradata

## Abstract

**Objective:**

We conducted a trial embedded within the German National Cohort comparing the responses to study invitations sent in recycled envelopes of grey color vs. envelopes of white color. We analyzed paradata for reactions to the invitation letters by potential subjects, the duration between mailing date of the invitation and active responses, and study participation.

**Results:**

Grey envelopes only slightly increased the chance of active responses (OR 1.16, 95% CI 0.83, 1.62) to the invitation letter. Potential study subjects with German nationality (OR 3.75, 95% CI 2.07, 7.66) and age groups above 50 years (50–59: OR 1.78, 95% CI 1.19, 2.69; 60–69: OR 2.25, 95% CI 1.48, 3.43) were more likely to actively respond to the invitation letter. The duration between mailing date of the invitation and active response was not associated with envelope color, sex, nationality, or age. Our trial replicates previous observations that the color of the envelope of a study invitation does not influence the likelihood of an active response or study participation.

**Electronic supplementary material:**

The online version of this article (10.1186/s13104-019-4510-y) contains supplementary material, which is available to authorized users.

## Introduction

In recent years, the problem of decreasing response in population-based research has received considerable attention [1–5] and although its implications are still a matter of debate [6–11], there seems to be a consensus that a higher response is generally preferable [10, 12]. A systematic review by Edwards and colleagues [12] reported that already some low-level characteristics of the delivery (e.g., recorded or first-class delivery, hand-written addresses) can increase the response to a mailed survey. The color of envelope (brown vs. white) did not influence the response [12] but may do so in other cultural contexts. We compared the response to grey vs. white envelopes that we used for invitations to a large cohort study in Germany. Grey envelopes are commonly used by German official authorities and we assumed that a more official character might influence the recipient’s attitude towards the contents of the letter. The response might also be influenced the by fact that grey envelopes apparently are made from recycled paper, whereas the paper source is not obvious for white envelopes.

In this trial, embedded in the German National Cohort (GNC, German: NAKO Gesundheitsstudie [13]), we investigated whether the envelope color of the first invitation influenced the probability of a reply to the invitation, the delay between mailing date and replies, and, finally, the probability of study participation.

## Main text

### Methods

The GNC is a cohort study investigating the causes for the development of major chronic diseases. The baseline examinations are conducted from 2014 to 2019. In 18 regional study centers across Germany, a random sample of the general population including a total of 100,000 women and 100,000 men aged 20–69 years will be examined. Potential study participants are randomly drawn from the regional registries of residents and corresponding contact details are provided to the respective study center. The baseline assessments include an extensive interview and self-completion questionnaires, a wide range of medical examinations and the collection of various biomaterials. Detailed information can be found elsewhere [13].

The recruitment protocol of the GNC includes an invitation letter, followed by up to three reminder letters separated by waiting periods of 14 days. The invitation letter asks potential participants to either return a pre-stamped response letter, e-mail, or to call the study center using a toll-free telephone number. For potential study subjects with known phone numbers, the invitation letter is followed by phone calls, and afterwards up to three reminder letters. The recruitment is controlled by MODYS [14], a dedicated software that schedules recruitment tasks and electronically documents all paradata, that is, detailed data about the recruitment process (e.g., events, attempted and successful contacts with potential subjects).

This trial was conducted in the Bremen study center of the GNC which will recruit a total of 10,000 cohort participants. The trial was restricted to potential subjects without known phone numbers to prevent phone calls of the study center during the waiting period.

For this trial, we planned to send out invitation letters with 1925 white and 1925 grey envelopes during 8 consecutive weeks between February and April 2017. With this sample size and an assumed response of approximately 11%, a response change of ± 3 percentage points can be detected with a power of 0.80. In each week letters were sent out on 2 days (usually Monday and Tuesday, according to the normal mailing schedule of the study) with only white envelopes used on 1 day and only grey ones on the other. Colors were randomly assigned to weekdays prior to the trial. The number of letters sent out per day varied between 225 and 250. Due to human error white envelopes were sent out on a “grey day” once, resulting in a final sample size of 2174 white and 1595 grey letters. The remaining recruitment adhered to the general recruitment protocol outlined above. We analyzed paradata for reactions by potential subjects during the first waiting period of 14 days (responses by mail, phone, e-mail, or personal contact in the study center) and derived the outcome ‘active response’ (0: not responded vs. 1: responded). For subjects actively responding within the first waiting period, we calculated the duration in days between the mailing of the invitation letter and their response. Whether or not subjects eventually participated in the GNC baseline examination defined the second outcome ‘participation’ (0: not participated vs. 1: participated). In our analyses, we included the variables sex (female vs. male), nationality (German vs. non-German), and age (categories: 20–29, 30–39, 40–49, 50–59, and 60–69 years), as provided by the registry of residents. To adjust for potential differences between weekdays, data from the 4 months preceding the trial was used to calculate pre-trial baselines for the likelihood of active responses and study participation separately for each weekday letters were sent out. Likewise, pre-trial baselines were calculated for the mean duration to respond to the invitation letter.

**Fig. 1:**
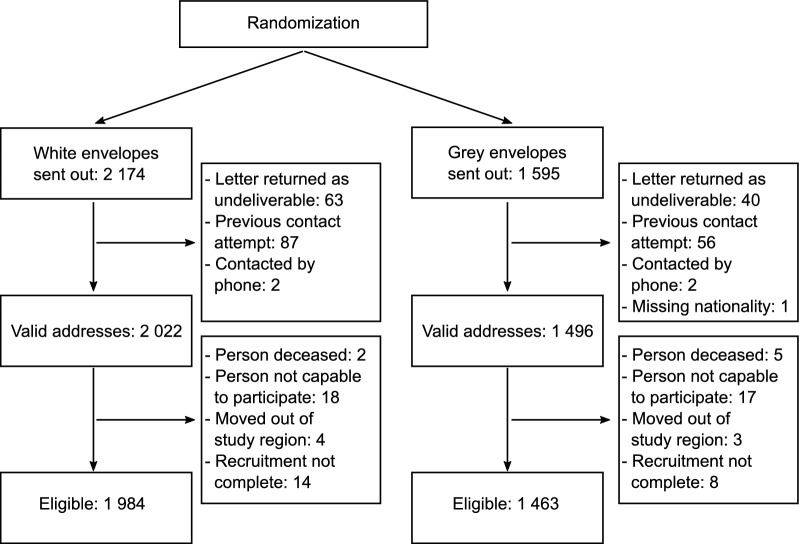
Consort flow chart

Subjects were excluded from further analyses if invitation letters were returned as undeliverable (i.e., subject moved or address turned out to be incorrect; N = 103) or if the paradata included recruitment events before the trial started (e.g., previous invitations sent to wrong addresses; N = 143) or phone calls initiated by the study center (N = 4). Furthermore, one subject was excluded because of missing data on nationality. The resulting analysis group consisted of 3518 subjects to whom letters with 2022 white and 1496 grey envelopes were sent (Fig. 1).

To estimate associations with the outcomes active response and participation we used logistic regression models adjusted for pre-trial likelihood of active responses and study participation to calculate odds ratios (ORs) and 95% confidence intervals (CIs). For the study participation model, 71 cases were excluded from the analysis (22 cases did not complete recruitment and 49 were not eligible, not capable, or deceased), reducing the analysis group to 3447 cases. We assessed the association between type of envelope and duration (ORs and 95% confidence intervals) to respond to the invitation letter with a linear regression model adjusted for pre-trial duration to respond to the invitation letter.

### Results

Only 171 subjects responded actively to the invitation letter while 373 eventually participated in the study. Grey envelopes only slightly increased the chance of active responses (OR 1.16, 95% CI 0.83, 1.62, Table 1). In contrast to non-Germans, potential study subjects with German nationality (OR 3.75, 95% CI 2.07, 7.66) and, compared to the age group 40–49, age groups above 50 years (50–59: OR 1.78, 95% CI 1.19, 2.69; 60–69: OR 2.25, 95% CI 1.48, 3.43) were more likely to actively respond to the invitation letter and also eventually to participate in the Bremen GNC study. Male and female subjects did not differ in their likelihood to actively respond (OR 0.73, 95% CI 0.53, 1.02) but males were less likely to eventually participate (OR 0.71, 95% CI 0.57, 0.90). For both outcomes, we checked whether envelope color interacted with age, sex, or nationality, but no meaningful interactions were found (results not shown).

**Table 1:** Response to Invitation and Participation in the Bremen Study Center of the German National Cohort

	Active response (N = 3518)					Participation (N = 3447^d^)				
	No		Yes^a^		OR^b^ (95% CI)	No		Yes		OR^c^ (95% CI)
	n	%	n	%		n	%	n	%	
Envelope color										
White	1929	95.4	93	4.6	1	1766	89.0	218	11.0	1
Grey	1418	94.8	78	5.2	1.16 (0.83, 1.62)	1308	89.4	155	10.6	0.95 (0.76, 1.19)
Sex^e^										
Female	1108	93.8	73	6.2	1	1003	86.2	161	13.8	1
Male	2239	95.8	98	4.2	0.73 (0.53, 1.02)	2071	90.7	212	9.3	0.71 (0.57, 0.90)
Nationality^e^										
Non-German	690	98.6	10	1.4	1	644	96.4	24	3.6	1
German	2657	94.3	161	5.7	3.75 (2.07, 7.66)	2430	87.4	349	12.6	3.46 (2.31, 5.43)
Age										
20–29	339	96.0	14	4.0	1.41 (0.73, 2.56)	319	93.3	23	6.7	0.89 (0.54, 1.41)
30–39	182	96.8	6	3.2	1.15 (0.43, 2.57)	177	95.7	8	4.3	0.58 (0.26, 1.15)
40–49	1321	96.9	43	3.2	1	1233	91.9	109	8.1	1
50–59	887	94.1	56	5.9	1.78 (1.19, 2.69)	784	85.0	138	15.0	1.83 (1.40, 2.40)
60–69	618	92.2	52	7.8	2.25 (1.48, 3.43)	561	85.5	95	14.5	1.67 (1.24, 2.25)
N	3347		171			3074		373		

*OR* odds ratio, *CI* confidence interval

^a^Includes subjects who replied by letter, phone call, e-mail or visited the study center in person

^b^Adjusted for pre-trial likelihood of active responses to invitation letter stratified by weekdays

^c^Adjusted for pre-trial study participation stratified by weekdays

^d^Subjects excluded from analysis (N = 71): recruitment not complete (N = 22), subject not eligible, not capable, or deceased (N = 49)

^e^Information provided by Bremen’s resident registration office

The duration between mailing date of the invitation and active response was not associated with envelope color, sex, nationality, or age (mean duration white envelopes 7.5 days vs. grey envelopes 7.4 days, Table 2).

**Table 2:** Duration between mailing date of the invitation and active response

	Mean	sd	ß^a,b^ (95% CI)
Envelope color			
White	7.5	3.1	0
Grey	7.4	3.2	0.65 (− 0.38, 1.68)
Sex^c^			
Female	7.4	3.0	0
Male	7.4	3.3	0.06 (− 0.92, 1.04)
Nationality^c^			
Non-German	8.2	3.2	0
German	7.4	3.2	− 0.56 (− 2.59, 1.47)
Age			
20–29	7.4	2.9	− 0.53 (− 2.45, 1.39)
30–39	6.8	2.8	− 0.66 (− 3.4, 2.08)
40–49	7.5	2.8	0
50–59	8.0	3.6	0.53 (− 0.71, 1.77)
60–69	6.8	3.1	− 0.74 (− 2.03, 0.54)
N	171		

*CI* confidence interval

^a^Includes subjects who replied by letter, phone call, e-mail or visited the study center in person

^b^Adjusted for pre-trial likelihood of active responses to invitation letter stratified by weekdays

^c^Information provided by Bremen’s resident registration office

### Discussion

Our trial replicates the observation by Edwards et al. [12] that the color of the envelope of a study invitation does not significantly influence the likelihood of an active response or study participation. An update of the two meta-analyses relevant to our study (Analyses 20.1. and 20.2. in [12]) resulted in only slightly decreased odds ratios and no changes to the authors’ original conclusions (Additional file 1: Figures S1, S2). Furthermore, our data confirm previous reports from the pretest of the GNC indicating that subjects with a foreign background are less likely to participate [15, 16]. It should be noted, that the execution of this trial was eased by the utilization of the MODYS software for recruitment, in which all measures of interest were routinely recorded. We would therefore advocate for the routine collection of paradata that would greatly facilitate the assessment of new trials or periodic replications of previous trials testing the effects of low-level or technical characteristics of recruitment schemes. In addition to dedicated software, however, collecting detailed paradata routinely of course also requires extra effort and diligence from the recruiting personnel, but once available, they offer opportunities for new insights on the recruitment process that would not be available without [14, 17].

## Limitations

It is not clear, however, whether the low response observed here generalizes to GNC as a whole since this trial is based only on a small sample from only one study center. Additionally, the sample in this trial excluded subjects with known phone numbers and phone contacts are known to have a positive effect on the response [15, 18].

## Additional file

**Additional file 1.** Results of the update of the meta-analysis conducted by Edwards and colleagues (2009) on the comparison of non-white vs. white envelope color on first response and final response.

## Data Availability

The datasets analysed during the current study are not publicly available due to privacy concerns but are available from the corresponding author on reasonable request.

## Abbreviations

CI: confidence interval
GNC: German National Cohort
OR: odds ratio
