# Supplementary material for: Study invitations with envelopes made from recycled paper do not increase likelihood of active responses or study participation in the German National Cohort
Source: BMC Res Notes. 2019 Jul 31;12:468. doi: 10.1186/s13104-019-4510-y (PMC6670214; doi:10.1186/s13104-019-4510-y)
Supplement: Supplementary file 1 — Additional file 1. Results of the update of the meta-analysis conducted by Edwards and colleagues (2009) on the comparison of non-white vs. white envelope color on first response and final response. [file 13104_2019_4510_MOESM1_ESM.docx]

**Additional file 1 to**

**Study invitations with envelopes made from recycled paper do not increase likelihood of active responses or study participation in the German National Cohort**

Malte Langeheine^1^, Hermann Pohlabeln^1^, Wolfgang Ahrens^1,2^, Kathrin Günther^1,*^, Stefan Rach^1,*,#^

^1^ Leibniz Institute for Prevention Research and Epidemiology - BIPS, Achterstrasse 30, D-28359 Bremen, Germany

^2^ Institute of Statistics, Faculty of Mathematics and Computer Science, University Bremen, P.O. Box 330 440, D-28334 Bremen, Germany

**Description: Results of the update of the meta-analysis conducted by Edwards and colleagues (2009) on the comparison of non-white vs. white envelope color on first response and final response including the results of Langeheine 2019.**


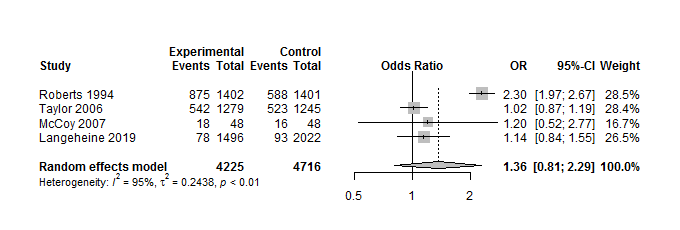


Figure S1 Comparison of non-white vs. white envelope, outcome first response


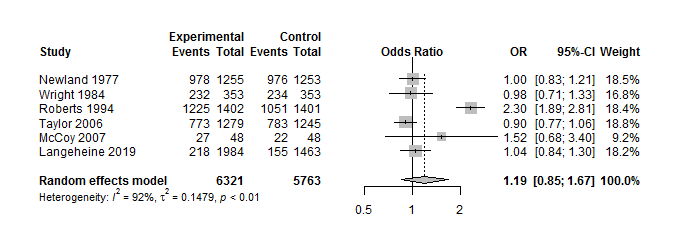


Figure S2 Comparison of non-white vs. white envelope, outcome final response

**References:**

Edwards PJ, Roberts I, Clarke MJ, Diguiseppi C, Wentz R, Kwan I, Cooper R, Felix LM, Pratap S: **Methods to Increase Response to Postal and Electronic Questionnaires.** *Cochrane Database Syst Rev* 2009**:**MR000008.
